# Heterogeneities in the case fatality ratio in the West African Ebola outbreak 2013–2016

**DOI:** 10.1098/rstb.2016.0308

**Published:** 2017-04-10

**Authors:** Tini Garske, Anne Cori, Archchun Ariyarajah, Isobel M. Blake, Ilaria Dorigatti, Tim Eckmanns, Christophe Fraser, Wes Hinsley, Thibaut Jombart, Harriet L. Mills, Gemma Nedjati-Gilani, Emily Newton, Pierre Nouvellet, Devin Perkins, Steven Riley, Dirk Schumacher, Anita Shah, Maria D. Van Kerkhove, Christopher Dye, Neil M. Ferguson, Christl A. Donnelly

**Affiliations:** 1 MRC Centre for Outbreak Analysis and Modelling, Department of Infectious Disease Epidemiology, Imperial College London, London W2 1PG, UK; 2 Big Data Institute, University of Oxford, Oxford OX3 7LF, UK; 3 MRC Integrative Epidemiology Unit, University of Bristol, Bristol BS8 2BN, UK; 4 Center for Global Health Research and Education, Institut Pasteur, Paris 75015, France; 5 WHO, 1211 Geneva, Switzerland; 6 Robert Koch Institute, 13302 Berlin, Germany

**Keywords:** Ebola virus disease, case fatality ratio, severity, mortality, outlier detection, spatial heterogeneity

## Abstract

The 2013–2016 Ebola outbreak in West Africa is the largest on record with 28 616 confirmed, probable and suspected cases and 11 310 deaths officially recorded by 10 June 2016, the true burden probably considerably higher. The case fatality ratio (CFR: proportion of cases that are fatal) is a key indicator of disease severity useful for gauging the appropriate public health response and for evaluating treatment benefits, if estimated accurately. We analysed individual-level clinical outcome data from Guinea, Liberia and Sierra Leone officially reported to the World Health Organization. The overall mean CFR was 62.9% (95% CI: 61.9% to 64.0%) among confirmed cases with recorded clinical outcomes. Age was the most important modifier of survival probabilities, but country, stage of the epidemic and whether patients were hospitalized also played roles. We developed a statistical analysis to detect outliers in CFR between districts of residence and treatment centres (TCs), adjusting for known factors influencing survival and identified eight districts and three TCs with a CFR significantly different from the average. From the current dataset, we cannot determine whether the observed variation in CFR seen by district or treatment centre reflects real differences in survival, related to the quality of care or other factors or was caused by differences in reporting practices or case ascertainment.

This article is part of the themed issue ‘The 2013–2016 West African Ebola epidemic: data, decision-making and disease control’.

## 1. Introduction

Ebola virus disease (EVD) is a viral haemorrhagic fever (VHF) and one of the most deadly human pathogens known, with reported case fatality ratios (CFRs) from previous outbreaks typically in the range 70–90% for the most severe Zaire strain that has caused the recent outbreak in West Africa [1], although a CFR as low as 44% (95% CI 26–62%) has also been reported [2]. During the 2013–2016 outbreak, 28 616 confirmed, probable and suspected cases and 11 310 deaths were officially reported to the World Health Organization (WHO) from the three most affected countries Guinea, Liberia and Sierra Leone by 10 June 2016 [3]. With large-scale transmission interrupted in all three countries the Public Health Emergency of International Concern was lifted on 29 March 2016 [3] despite the fact that one might expect further resurgences, for instance due to low-level unobserved ongoing transmission or incomplete clearance or reactivation of the virus in some survivors.

Throughout this outbreak estimates of the CFR have varied widely, depending on the sub-population studied and the methods employed [4–14]. Early estimates often simply estimated CFR by using the number of deaths divided by the number of cases reported to date [15,16]. These estimates did not consider the fact that in a growing outbreak a significant proportion of reported cases may yet die, or the fact that the clinical outcome is only reported in a fraction of cases, therefore under-estimating the true CFR [17,18]. While estimates of the CFR stemming from analysis of the whole outbreak have been in line with those reported from previous outbreaks, typically around 60–70% [11,12,19], CFR estimates from smaller studies have been more variable and often considerably lower [4–10,13], owing to the fact that the study populations were not representative of the overall characteristics of reported cases, for instance often focusing on hospitalized cases only.

Understanding factors influencing mortality is important to not only understand the severity of a pathogen and factors associated with differences in mortality, but also to improve patient care. For example, the evaluation of the efficacy of treatment regimens or new drugs relies on accurate baseline CFR estimates, and study design of randomized controlled trials can be adapted to take into account important determinants of patient outcomes [7]. A strength of small, patient-based studies to investigate determinants of the CFR are the often well-defined inclusion criteria and comparability between different study arms or strata. This may allow for an assessment if not adjustment of biases in CFR estimates resulting from the study design. However, the limited scope can make it impossible to detect some of the factors influencing mortality, and therefore a bird's eye view of the situation is crucial to fully capture the observed heterogeneities.

Here, we estimate the CFR of EVD in the West African outbreak from a comprehensive individual-based dataset of cases reported officially to the WHO and we investigate factors modifying the CFR. We assess spatial heterogeneity in mortality by stratifying CFR estimates by district and treatment centre (TC) and identify outliers of significantly lower or higher CFR than expected. This enhances the understanding of drivers of mortality and highlights which districts or TCs could be investigated further to understand the reasons for the unusual outcome composition recorded there.

## 2. Material and methods

### (a) Dataset

We analysed the VHF database of cases reported officially to the WHO during the 2013–2016 Ebola outbreak in Guinea, Liberia and Sierra Leone as previously described [11], updated on the 28 September 2015 including updates from Guinea on 27 September, from Liberia on 4 May and from Sierra Leone on 28 September. This dataset included data on individual cases using a ‘VHF case investigation form’ recording (among a large number of other variables) clinical outcome, epidemiological case classification, demographic information such as age and gender, location of residence, hospitalization status (i.e. whether cases were hospitalized), and TC as well as dates of case report, symptom onset, hospitalization, death or recovery and outcome report. We performed extensive data cleaning as previously described [11,12]. Location of residence was geo-coded to the second administrative level, termed prefecture in Guinea, county in Liberia and district in Sierra Leone. For conciseness, we refer to this as district for all countries throughout. For cases with missing date of symptom onset, we inferred a likely date based on other dates recorded, such as the dates of report or hospitalization, and the median delay observed between the events in question in the current dataset, following methods developed earlier [11]. We publish the cleaned version of this dataset as electronic supplementary materials 1 and 2, including a subset of the recorded variables that are necessary to perform the analyses presented here.

We estimated the CFR as the percentage of fatalities among cases with reported definitive clinical outcome (dead/alive). As in previous analyses of the CFR [11,12,20,21], we only considered cases which were reported prior to their clinical outcome being known, as cases reported retrospectively were heavily biased towards fatal outcomes through case detection associated with burial teams finding bodies. We therefore excluded any cases for which the date of report was on or after the date of outcome recording, if both dates were given. We refer to this as ‘retrospective reports’ throughout.

Individuals in the dataset were classified into five epidemiological case classifications: confirmed, probable, suspected, not a case, and excluded. Official case definitions were based on clinical symptoms and testing [22], though interpretations probably differed between countries, with the most complete and consistent recording among confirmed cases across the three countries. While the overall extent of the epidemic is likely best described using a fairly inclusive case definition with probable and perhaps suspected cases included, here we focus exclusively on confirmed cases to obtain less-biased estimates of the CFR by restricting to the most reliably recorded subset of cases despite the resulting smaller sample size. However, as a sensitivity analysis we also present the main results having included both confirmed and probable cases in electronic supplementary material 3.

We classified TCs broadly into five types: Ebola Treatment Unit (ETU), Hospital, Holding Centre, Community Care Centre (CCC), Health Centre, and added a category for those of unknown type, and two further categories for patients never hospitalized or of unknown hospitalization status (for further details see the text box 1 and table S1 in electronic supplementary material 3). We refer to those who were recorded in any of the five TC types as ‘hospitalized cases’ throughout.

Box 1. **Treatment centre classification.**

We classified treatment centres (TCs) broadly into five types:

1. Ebola Treatment Units (ETUs)—purpose-built or repurposed buildings designated for the care and management of Ebola patients. These admitted patients with suspected EVD or with confirmed EVD referred from other healthcare units, although the admission and referral procedures varied between facilities.
2. Hospitals—hospital wards in existing hospitals. Some hospitals had isolation facilities for suspected or confirmed EVD patients.
3. Holding Centres—basic facilities to isolate suspected EVD patients until laboratory confirmation, following which patients should be transferred to an ETU if beds were available.
4. Community Care Centres (CCCs)—set up to alleviate pressure from ETUs by providing basic care and isolation to suspected EVD patients. Staffing levels were lower than in ETUs, and sometimes relatives provided care within the CCC setting.
5. Health Centres—we classified a variety of healthcare facilities as Health Centres, including those described as Case Management Centre, Clinic, Health Centre, Health Post, Health Unit, Maternal and Child Health Post or Referral Centre.

### (b) Analyses

#### (i) Identifying individual-level predictors of mortality

We investigated the extent to which variables could explain clinical outcome by calculating stratified CFR estimates and performing *χ*^2^-tests, and using uni- and multivariable logistic regression models.

We calculated the CFR stratified by TC type and country and performed pairwise comparisons between strata (i.e. 21 comparisons), assessing significance using *χ*^2^-tests and implementing a Bonferroni correction [23] to adjust for multiple testing.

We investigated the effect of the delay from onset to hospitalization on clinical outcome by performing a *χ*^2^-test between patients hospitalized within 3 days of symptom onset to those hospitalized later. We furthermore fitted a univariable logistic regression model between clinical outcome and the delay from onset to hospitalization.

To describe the age dependence with the clinical outcome, we fitted logistic regression models with age as a trend, separately for individuals under 15 years and individuals 15 years and older (here referred to as children and adults, respectively) to capture the observed age dependence, by defining child age *a*_child_ and adult age *a*_adult_ as separate variables,

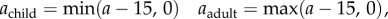
where *a* is the age in years, and including these in the regression.

Date of onset was aggregated into quarters and investigated both as a linear trend and as a categorical variable, while we investigated both hospitalization status (recorded as yes, no or missing), and TC type as categorical variables using logistic regression. For all categorical variables, we chose the largest category as the reference.

We fitted multivariable logistic regression models using all possible combinations of the covariates that were significantly associated with clinical outcome in univariable regression. For date of onset, we tested both inclusion as a trend or as a categorical variable. We chose the model with minimal value of Akaike's Information Criterion (AIC) [24] as the most parsimonious multivariable regression model to avoid overfitting and to only include variables that were independently associated with the outcome.

#### (ii) Assessing heterogeneity in district- and treatment centre-specific case fatality ratio

We assessed heterogeneities in CFR between districts and TCs following methods used to identify outlier healthcare units from routinely collected outcome data in the UK National Health Service [25]. To evaluate whether variability in CFR estimates was larger than would be expected by chance we plotted district- and TC-specific estimates against their sample sizes in funnel plots [26], highlighting the range of variation expected under a binomial process with the same overall mean by also plotting relevant quantiles of the binomial distribution with this mean for each sample size.

The case mix, i.e. the composition of the cases in terms of the covariates considered in these analyses, such as age and onset date, differed between districts and between TCs. We adjusted for case mix in individual districts or TCs based on the variables identified in the best-fitting multivariable regression models when fitted to the subset of cases for which the district of residence was recorded, or which were hospitalized with the TC recorded, respectively. For this, we calculated model predictions (i.e. the probability of death) for all cases based on their covariates, and averaged over all cases within a district or TC to obtain the expected CFR. Here, we excluded cases from districts or TCs with less than 10 cases. To avoid undue influence of potential outliers on the results, we used a full cross-validation approach by refitting the model omitting each district or TC in turn and generated model predictions (i.e. the probability of death) for the omitted TC. We then simulated outcomes for these cases based on the model predictions, resampling each district or TC *n*_sim_ = 250 000 times, and compared the observed number of deaths with the distribution of simulated outcomes to evaluate the probability of a result at least as extreme as the observed number of deaths among cases, evaluating the proportion *p* of these realizations that resulted in a CFR lower than the observed. The raw *p*-value for a significantly lower than expected CFR was evaluated as *p*_low_ = 2*p*/*n*_sim_, while the raw *p*-value for a significantly higher than expected CFR was *p*_high_ = 2(1 − *p*)/*n*_sim_. We adjusted these raw *p*-values for multiple testing using a Bonferroni correction.

## 3. Results

In contrast with the 28 616 cases quoted in WHO situation reports, there were a total of 33 338 confirmed, probable and suspected cases reported by 28 September 2015 recorded in the VHF database analysed here (table 1; electronic supplementary materials 1 and 2). For evaluating the CFR, we only considered the 8413 confirmed cases with known clinical outcome that were reported prospectively rather than retrospectively (tables S2 and S3 in electronic supplementary material 3). As expected, cases reported retrospectively were heavily biased towards corpses found post-mortem, and indeed the CFR among these was much higher at 91.3% (95% CI 89.5–92.9%) among confirmed cases from all three countries together (figure S1 in electronic supplementary material 3).

**Table 1.** Number of cases by country and epidemiological case definition, including retrospectively reported cases, and percentage of retrospectively reported cases (which were excluded from further analysis), among confirmed cases.

	all countries	Guinea	Liberia	Sierra Leone
confirmed	16 444	3304	3743	9397
probable	3910	443	1600	1867
suspected	12 984	10	2787	10 187
total	33 338	3757	8130	21 451
% reported retrospectively	6.5	14.9	4.4	4.3

**Figure 1.**
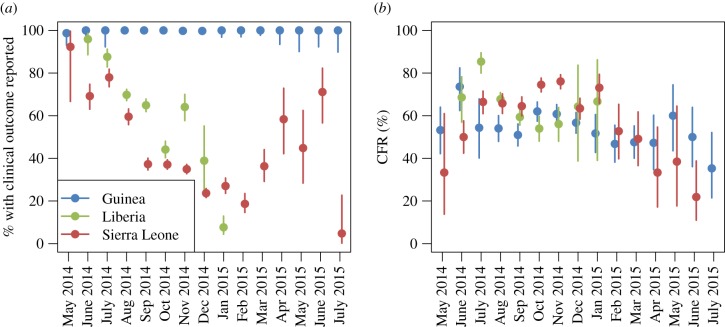
(*a*) Percentage of confirmed cases with reported definitive clinical outcome. (*b*) Estimated CFR, both with 95% confidence interval (CI) by country and month of onset (inferred). Only data for months with 10 or more confirmed cases (in the particular country) are shown.

Completeness of reporting the clinical outcome was highly variable between countries, epidemiological case definitions and over time (figure 1, as well as figures S3 and S5 in electronic supplementary material 3), with outcomes more likely reported in confirmed cases overall, while CFR estimates were lower and more consistent between countries in confirmed cases than in probable or suspected cases.

In Guinea, the outcome was reported in nearly all confirmed cases, while in Liberia and Sierra Leone reporting rates of clinical outcome dropped drastically during the peak of the epidemic (August–December 2014) when healthcare systems were at their most overstretched.

Other variables investigated, including date of symptom onset, age, district of residence, and, for hospitalized cases, TC and the onset to hospitalization delay, were recorded more completely (figure S2 in electronic supplementary material 3), with around 20% of onset dates missing. For nearly all cases with missing symptom onset date, we were able to infer the likely onset date based on other recorded dates (such as the date of case report). Age and district of residence were recorded in around 95% of cases, while the hospitalization status was only recorded for 75% of patients. Among those hospitalized, the particular TC was missing in around 20% of patients and both TC type and the onset to hospitalization delay were missing for around a quarter of patients.

**Figure 2.**
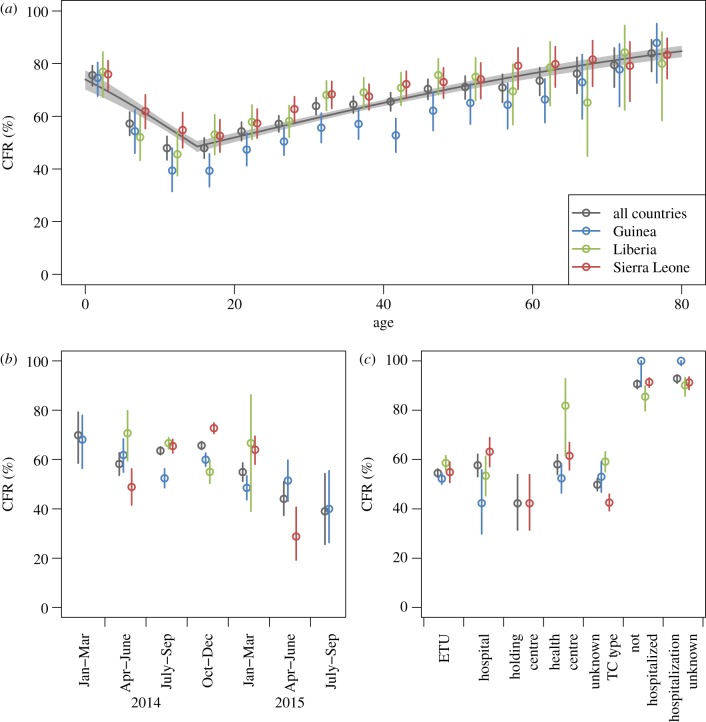
CFR by (*a*) age in 5-year age bands, (*b*) quarter, (*c*) Treatment Centre type, for all countries combined and by country for confirmed cases, with 95% CI. Only strata with 10 or more confirmed cases are shown. Note that there were no Community Care Centres with 10 or more cases with reported clinical outcome. (*a*) The solid black line shows the prediction from a logistic regression model with age fitted as a linear trend in children and adults separately with 95% CI (grey area), shown for all countries combined to keep the figure simple. Country-specific fits are shown in figure S6 in electronic supplementary material 3.

The overall CFR among the 8413 confirmed cases in the three countries for which the clinical outcome was reported was 62.9% (95% CI 61.9–64.0%). Several factors were associated with the observed CFR, including age, the epidemic stage (defined by the quarter of the year) and hospitalization status (figure 2; see also figure S7 in electronic supplementary material 3 for absolute frequencies of the covariates considered). The reported delay from symptom onset to hospitalization was not associated with the clinical outcome (*p* = 0.21 in a *χ*^2^-test and *p* = 0.68 when fitted as a trend in a logistic regression model).

Age had a profound effect on survival. The CFR in children was highest in children under the age of 5 at 75.6% (95% CI 71.6–79.2%), decreased with age throughout adolescence, until it reached a minimum at 47.9% (95% CI 45.0–50.8%) in the teenage age groups from 10 to 19 years, and then increased steadily with age with the highest rates at 83.9% (95% CI 77.0–89.0%) in the elderly over the age of 75 in the three countries overall. This pattern was consistent across all three countries. A logistic regression model treating the effect of age as a trend separately in children and adults captured the observed pattern very well (*p* < 0.001; see figure 2*a*; figure S6 in electronic supplementary material 3), although there was substantial heterogeneity in the data that was not explained by this simple model. Allowing country-specific differences in the overall levels of CFR was a significant model improvement (*p* < 0.001), but the age trends were not significantly different between countries (*p* = 0.11).

The estimated CFR varied throughout the epidemic, with a decreasing trend overall, from 69.8% (95% CI 58.6–79.2%) between January and March 2014 to 39.0% (95% CI 25.7–54.3%) between July and September 2015 for all countries combined (figure 2*b*). This was driven by the decreases in Guinea and Liberia, while the high CFR during the peak with lower values early and late in the epidemic in Sierra Leone mirrored the pattern of clinical outcome reporting and meant that fitting the quarter of onset as a categorical variable improved model fit significantly, whether country was included as additional factor or not (*p* < 0.001 without and with country). Including country into the model was also a significant improvement (*p* < 0.001).

TCs of unknown type had a significantly lower CFR at 49.8% (95% CI 47.4–52.2%) than ETUs (54.5%, 95% CI 52.9–56.1%, *p* = 0.033) and Health Centres (58.0%, 95% CI 54.0–62.0%, *p* = 0.015). Those not hospitalized (CFR 90.6%, 95% CI 88.7–92.2%) or of unknown hospitalization status (CFR 92.8, 95% CI 91.0–94.3%) had a significantly higher CFR than those hospitalized in any of the TC types (all *p* < 0.001; figure 2*c*).

The best-fitting multivariable regression model (selected based on the minimal AIC value) included age, country, inferred date of symptom onset (as a categorical variable for district-based analysis and as a linear trend for TC-based analysis) and TC type (for model parameters see tables S4 and S5 in electronic supplementary material 3). The district-based analysis included 8034 confirmed cases for which the district, the clinical outcome and the considered covariates were recorded, while the TC-based analysis only included a total of 4865 hospitalized cases with the required data recorded. This smaller underlying dataset resulted in lower statistical power, and therefore the AIC favoured a slightly simpler model fitting the date of symptom onset as a linear trend rather than as a categorical variable (as in the district-based analysis).

**Figure 3.**
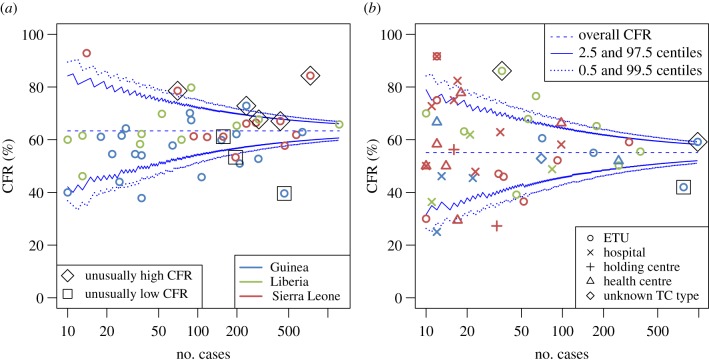
Funnel plot of the CFR by (*a*) district of residence and (*b*) TC, for confirmed cases. Only districts and TCs with 10 or more confirmed cases are included. Districts and TCs with significantly high or low CFR after adjusting for covariates are marked with diamonds and squares, respectively. Blue solid and dotted lines show the 95% and 99% binomial confidence intervals, respectively, for each sample size assuming the mean CFR of the patients included in these analyses (dashed blue line).

Based on these models, we predicted the expected CFR for each district or TC, respectively, and compared the predictions with the observed CFR. Figure 3 shows the district- or TC-specific CFR estimates plotted against the number of reported confirmed cases for each district or TC. In the absence of any significant differences, we would expect larger variation around the overall mean (63.4% (95% CI 62.3–64.4%) and 54.4% (95% CI 52.9–55.8%) for the district- and TC-based analyses, respectively) for small than for large sample sizes as indicated by the funnel. The saw-tooth appearance of the funnel stems from the fact that sample size is discrete, with the expected number of deaths increasing at approximately every other step in sample size for a grand mean CFR in the order of 50%, leading to small fairly regular fluctuations in the plotted quantiles as a function of the sample size. There was considerable variation beyond what would be expected by chance, both between districts and between TCs, with 14 of 43 districts and 18 of 66 TCs falling outside the 95% funnel (see also figure S8 in electronic supplementary material 3).

We adjusted for case mix within each district or TC based on the multivariable regression models to compare individual observed CFRs to what would be expected for the particular district or TC, rather than the grand mean among all cases included in these analyses. We identified eight districts of residence and three TCs with a CFR significantly different from the expected (tables 2 and 3). Three districts had significantly lower and five significantly higher CFR than expected, and some but not all of these fell outside the funnel that indicates the expected variation from the overall CFR for any particular sample size, not accounting for case mix. Case mix explained even more of the variability in CFR between TCs, with only three TCs identified as outliers. The two TC outliers in Guinea were in districts that were also identified as outliers. This is not surprising as the majority of cases from these districts were hospitalized in these local TCs, although both TCs cared for substantial numbers of cases from outside the district also.

**Table 2.** Districts with significantly lower or higher CFR among confirmed cases than expected.

country	district	*N*	observed CFR (95% CI)	expected CFR^a^ (95% CI)	direction	*p*-value
Guinea	Conakry	462	39.6 (35.3–44.1)	54.6 (50.1–59.1)	low	<0.001
Sierra Leone	Moyamba	195	53.3 (46.3–60.2)	66.9 (60–73.1)	low	<0.001
Sierra Leone	Kambia	157	61.1 (53.3–68.4)	79.3 (72.3–84.9)	low	<0.001
Guinea	Gueckedou	236	72.9 (66.9–78.2)	56.2 (49.8–62.4)	high	<0.001
Sierra Leone	Western	733	84.3 (81.5–86.8)	74.2 (70.9–77.2)	high	<0.001
Liberia	Lofa	294	67.7 (62.1–72.8)	57.6 (51.9–63.1)	high	0.0082
Sierra Leone	Tonkolili	70	78.6 (67.6–86.6)	60.7 (49–71.3)	high	0.011
Sierra Leone	Kenema	431	67.1 (62.5–71.3)	61.2 (56.5–65.7)	high	0.023

^a^Expected based on case mix adjusting for country, age, quarter of onset (fitted as a categorical variable) and TC type.

**Table 3.** Treatment centres with significantly lower or higher CFR among confirmed cases than expected.

country	treatment centre^a^	*N*	observed CFR (95% CI)	expected CFR^b^ (95% CI)	direction	*p*-value
Guinea	Conakry 2	774	42 (38.6–45.5)	54.9 (51.4–58.4)	low	<0.001
Guinea	Gueckedou 1	982	59.2 (56.1–62.2)	46.7 (43.6–49.8)	high	<0.001
Liberia	Montserrado 65	36	86.1 (71.3–93.9)	59.5 (43.3–73.8)	high	0.0023

^a^Treatment centre names were anonymized within district.

^b^Expected based on case mix adjusting for country, age, quarter of onset (fitted as trend) and TC type.

## 4. Discussion

In this study, we evaluated the CFR in the 2013–2016 Ebola outbreak in West Africa, using individual-level clinical outcome data, which allowed us to investigate the effects of several covariates on outcomes. As found in earlier analyses, CFR varied by age (with lowest CFRs found in teenagers, and higher CFRs in young children as well as increasing with age in adults), over time (with a decreasing trend in Guinea and Liberia, but highest values during the peak around October 2014 in Sierra Leone) and by hospitalization status, i.e. whether or not cases were hospitalized (with hospitalized cases significantly less likely to die) [11,12,20,21,27]. We found considerable spatial heterogeneities in CFR between districts and TCs, some of which we explained with the available covariates, and identified outliers among districts and TCs for which the observed CFR was significantly different from what would be expected after adjusting for the case mix based on the identified covariates.

Despite the very simple definition of the CFR and its importance for public health planning, it is surprisingly difficult to estimate it accurately in outbreak situations as the data are frequently collected under challenging circumstances and not specifically gathered for this purpose, which can lead to potentially substantial biases [18]. The main biases that might affect the validity of the present study stem from incomplete reporting at two levels.

Firstly, incomplete case ascertainment can lead to biases in estimates of the CFR if the reasons for cases being officially reported are correlated with the outcome, for instance when more severe cases are more likely to be reported. In the 2013–2016 Ebola outbreak substantial underreporting rates were estimated [28,29]. There were several mechanisms for cases to be entered into the database, including through notification via phone hotlines, at hospitalization, through contact tracing and burial teams. While cases found through contact tracing may be less biased with regards to severity, both notification via hotlines or through hospitalization may be somewhat biased towards more severe cases as these may be more likely to seek care, while on the other hand for a disease as severe as Ebola the patients reaching a TC before dying may be a sample that is skewed towards survivors. Burial teams would likely primarily identify cases found post-mortem and therefore this subset is likely highly biased towards death. Unfortunately, no information on the mechanisms by which individual cases enter the database was recorded, and it is therefore not possible to assess the impact this has on CFR estimates. Recording this type of information would be a valuable addition to data collection and should be included routinely in any outbreak investigation. Here, we aimed to reduce the most severe bias associated with underreporting by excluding cases for which the clinical outcome was already known on the date of reporting, as these were dominated by cases found by burial teams.

Secondly, reporting of the definitive clinical outcome was incomplete with data missing from nearly half of the cases in the database. Outcome reporting rates in confirmed cases were near complete in Guinea, but less than 40% in Sierra Leone. Clearly, if the probability of the clinical outcome report differed between survivors and fatalities, the large amount of missing data could also lead to substantial biases in CFR estimates. For instance, it is plausible that deaths may be more likely recorded than survival as the average delay from onset to death is shorter than from onset to recovery, therefore loss to follow up may occur more frequently among survivors than fatalities. Adjusting for these biases may be possible to some extent and is a subject of ongoing research. However, it is reassuring that CFR estimates based on confirmed cases with reported outcome were fairly consistent between countries, despite the differences in countries' reporting practices.

Despite the biases associated with problems in reporting, we found age to be a clear modifier of survival probability, with very high values of the CFR in young children (less than 5 years; figure 2*a*), decreasing CFR with increasing age to a minimum at around 15 years, and then increasing with age to very high values in the elderly. While this effect has been observed in other studies [4,7,10,20,30,31], the large sample size available here gave a very clear picture of the age dependence, which we fitted well with a simple two-parameter model. The fact that despite obvious differences in reporting practices between countries the age trends are very similar across all three countries gives confidence that this is in fact a real biological effect.

We also found differences in CFR between hospitalized and non-hospitalized cases [11,12], with extremely high CFRs of around 90% for those not hospitalized or of unknown hospitalization status, and much lower values in the range of 40 to 60% for hospitalized cases. Naively, this could be interpreted as substantial benefit of clinical care. However, the biases in both case and outcome reporting are likely to result in lower CFR estimates in hospitalized than non-hospitalized cases. In particular, follow up and therefore reporting of the definitive clinical outcome were likely less complete in the community setting, resulting in estimates with a stronger bias towards death for those cases. Furthermore, delays to hospitalization could also result in a higher CFR in the community setting compared with hospitalized patients as the most severe cases may die very quickly, sometimes before they had the opportunity to reach healthcare facilities due to long distances and limited transport, skewing the sample of hospitalized cases towards survivors [32]. While we have found no association between the delay from symptom onset to hospitalization and clinical outcome, initial analyses indicate that such effects could explain the observed difference in CFR between TC and community settings. While the difference in CFR estimates between hospitalized and non-hospitalized cases is striking, caution should be taken when interpreting this result as we cannot yet quantify the magnitude of the biases of CFR estimates in the community setting or disentangle the contributions from the different mechanisms that may give rise to genuine differences in CFR. Further work is needed to assess the magnitude of the remaining biases.

By contrast, among the more homogeneous population of hospitalized patients, the observed differences in CFR between TC types were fairly small and mostly non-significant. While early hospitalization of cases has been found to be associated with reduction in transmission intensity [33], the delay from onset to hospitalization (among hospitalized patients) was not significantly associated with clinical outcome in this study. Individual patients' viral load has been found to be a strong predictor of survival [7–9]; however, we cannot investigate this finding with the VHF dataset due to data limitations.

The time trends in CFR differed between countries as previously reported [27], with a decreasing trend found in Guinea and Liberia, but higher values during the peak than early or late in the Sierra Leonean epidemic. The decreasing trend in Guinea and Liberia is encouraging, while the high CFR values during the peak from August to December 2015 in Sierra Leone could reflect genuinely higher mortality during this most challenging part of the epidemic. However, this could equally be caused by preferential reporting of fatal outcomes, given that outcome reporting rates were lowest during this period in Sierra Leone. Given the incompleteness of outcome reporting, any such biases clearly have the potential to influence the CFR estimates, and further work is needed to quantitatively assess this effect on CFR estimates.

We examined whether outliers in CFR among districts of residence or TCs could be identified, recognizing that the threshold for doing so is higher than for just detecting greater than expected variation across all districts or TCs. We found considerable variation in both district- and TC-specific CFRs over and above what would be expected by chance. For districts, we could explain some of this variation by allowing for case mix in terms of country, age, date of symptom onset (fitted as a categorical variable) and TC type. Even after the adjustment, there were a considerable number of districts with significantly lower or higher CFR than would be expected for the case mix reported from each district. However, these were different districts to those that would be identified based on comparing the individual CFR to the overall mean, showing the importance of the covariates identified here.

On the other hand, we were able to explain the majority of the variation in CFR between TCs by adjusting for covariates including age, country, date of onset (here fitted as a linear trend rather than a categorical variable) and TC type, indicating that standards of clinical care may not have differed enough between TCs to affect patient outcomes. This could be either because they were fairly homogeneous between TCs, or because the levels of clinical care available in the resource-poor West African setting were simply not sufficient to make much of a difference. This is also plausible in the context of little differences between TC types, but would be in contrast with the experience with cases among western healthcare workers who were med-evacuated and cared for in state-of-the-art high dependency units that resulted in considerably lower mortality [34,35]. However, the few outliers identified would be good candidates for further selective investigation, to identify the characteristics that set these apart, based on complementary datasets, e.g. from MSF (Médicins Sans Frontières). In particular, it could be extremely informative to compare data on referral routes into each ETU to explain the remaining variability in CFR between ETUs as one might expect that confirmed cases referred from other healthcare units would have higher survival chances at the point of referral (as they had probably survived for several days already) than those admitted early on as suspected cases.

This epidemic was the largest outbreak of EVD in history by two orders of magnitude, and overwhelmed the fragile healthcare systems in the affected countries. Local and international agencies mounted an enormous public health response in logistically challenging settings. While the focus during this time clearly was on patient care and the interruption of transmission, the epidemiological dataset collected throughout is extremely detailed and rich despite its shortcomings. The biases stemming from the mechanisms of data collection mean that thoughtful analysis is needed and results must be interpreted with care. However, the large sample size and regional scope allowed us to investigate patterns more thoroughly than would be possible otherwise.

## Supplementary Material

Database of Ebola Virus Disease Cases

## Supplementary Material

Data Dictionary

## Supplementary Material

Further Details and Sensitivity Analyses.

## Data accessibility

The datasets supporting this article have been uploaded as part of the electronic supplementary material.
