# Supplementary material for: Heterogeneities in the case fatality ratio in the West African Ebola outbreak 2013–2016
Source: Philos Trans R Soc Lond B Biol Sci. 2017 Apr 10;372(1721):20160308. doi: 10.1098/rstb.2016.0308 (PMC5394646; doi:10.1098/rstb.2016.0308)
Supplement: Further Details and Sensitivity Analyses. [file rstb20160308supp3.pdf]

Heterogeneities in the Case Fatality Ratio in the West African Ebola Outbreak 2013 – 2016

Electronic Supplementary Material 3: Further Details and Sensitivity Analyses

Tini Garske[1], Anne Cori[1], Archchun Ariyarajah[5], Isobel M Blake[1], Ilaria Dorigatti[1], Tim Eckmanns[5,6], Christophe Fraser[1,2], Wes Hinsley[1], Thibaut Jombart[1], Harriet L Mills[3], Gemma Nedjati-Gilani[1], Emily Newton[5], Pierre Nouvellet[1], Devin Perkins[5], Steven Riley[1], Dirk Schumacher[5], Anita Shah[5], Maria D Van Kerkhove[1,4], Christopher Dye[5], Neil M Ferguson[1], Christl A Donnelly[1*]

[1]MRC Centre for Outbreak Analysis and Modelling, Department of Infectious Disease Epidemiology, Imperial College London, UK
[2]Big Data Institute, University of Oxford, UK
[3]MRC Integrative Epidemiology Unit, University of Bristol, UK
[4]Center for Global Health Research and Education, Institut Pasteur, Paris, France
[5]WHO, Geneva, Switzerland
[6]Robert Koch Institute, Berlin, Germany
**Corresponding author*

Table of Contents

Treatment centre classification 2

Exclusion of retrospective case reports 2

Missing data 4

Effect of epidemiological case classification on outcome reporting and CFR estimates 5

Further results for the multivariable regression analysis 8

Sensitivity Analysis: Main results for confirmed and probable cases 12

Treatment centre classification

Our dataset contained 66 TCs with 10 or more confirmed cases (Supplementary Table 1).

Supplementary Table 1: Number of TCs by type and country.

	Guinea	Liberia	Sierra Leone
ETU	4	9	9
Hospital	3	4	10
Holding Centre	0	0	2
Community Care Centre	0	0	1
Health Centre	2	0	13
Unknown TC Type	2	0	7

Exclusion of retrospective case reports

Overall, 14.5% of cases were reported retrospectively, however, their proportion differed between countries and epidemiological case definitions (Supplementary Table 2 and Supplementary Table 3), with the lowest proportion of cases reported retrospectively in Liberia, followed by Sierra Leone and Guinea. The CFR in cases reported retrospectively was above 80% in all countries and for all epidemiological case classifications. This was considerably higher than that of cases reported before their outcome was known, confirming the expected bias. The exclusion therefore reduced bias in the estimated CFRs, leading to slightly lower estimates (Supplementary Figure 1).

Supplementary Table 2: Number of confirmed, probable and suspected cases with reported clinical outcome / total number of confirmed, probable and suspected cases reported by country and epidemiological case definition, excluding cases reported retrospectively.

	All countries	Guinea	Liberia	Sierra Leone
confirmed	8,413 / 15,378	2,805 / 2,812	2,223 / 3,577	3,385 / 8,989
probable	1,176 / 3,256	154 / 154	611 / 1,458	411 / 1,644
suspected	4,255 / 9,874	0 / 10	1,261 / 2,551	2,994 / 7,313
Total	13,844 / 28,508	2,959 / 2,976	4,095 / 7,586	6,790 / 17,946

Supplementary Table 3: Percentage of confirmed, probable and suspected cases reported retrospectively by country and epidemiological case definition.

	All countries	Guinea	Liberia	Sierra Leone
confirmed	6.5	14.9	4.4	4.3
probable	16.7	65.2	8.9	11.9
suspected	24.0	0.0	8.5	28.2

© The Authors under the terms of the Creative Commons Attribution License http://creativecommons.org/licenses/by/3.0/, which permits unrestricted use, provided the original author and source are credited.

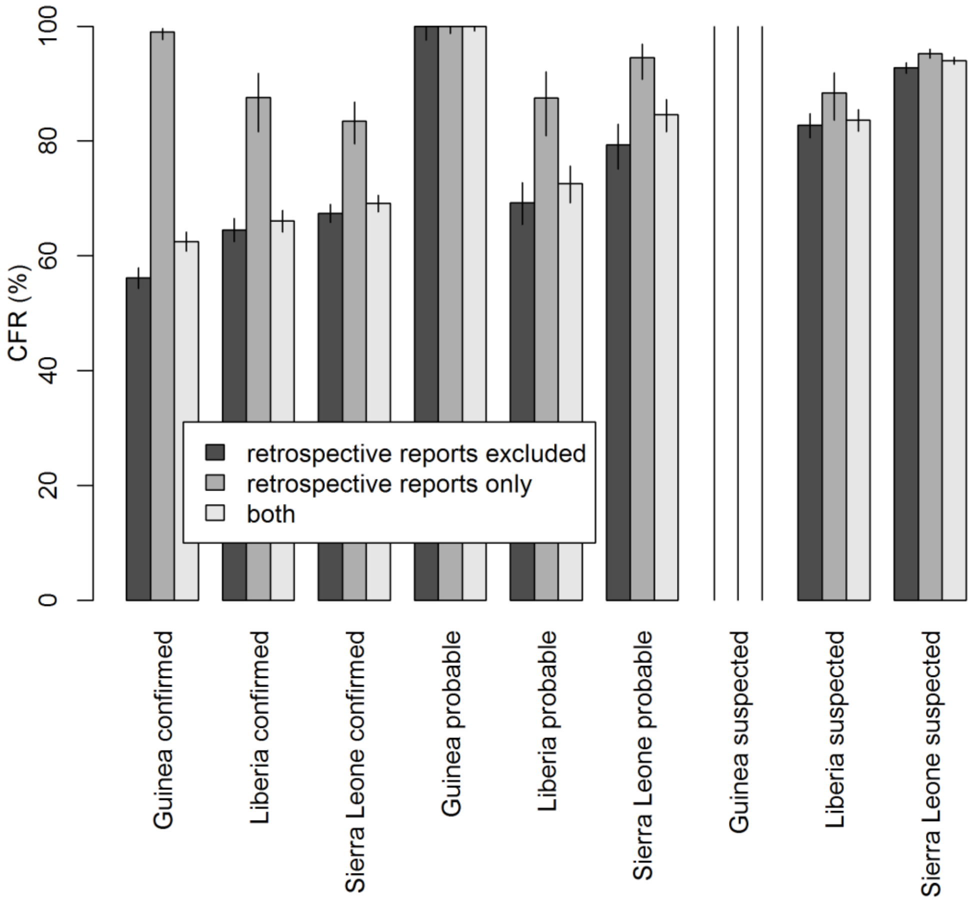

Supplementary Figure 1: Estimated CFR by country, case definition and timing of clinical outcome reporting, highlighting the effect of excluding cases reported retrospectively on CFR estimates.

© The Authors under the terms of the Creative Commons Attribution License http://creativecommons.org/licenses/by/3.0/, which permits unrestricted use, provided the original author and source are credited.

Missing data

Of the variables analysed here, the clinical outcome was the least completely recorded, with very high levels of reporting in age and district of residence. For nearly all cases with missing onset dates we could infer the onset date based on alternative dates recorded. Variables relating to hospitalisation had a lower denominator as these were only relevant for hospitalised cases. They were missing in around 20 to 25% of hospitalised cases (Supplementary Figure 2). There was a marked difference in completeness between the three countries, with data recorded much more completely in Guinea than the other countries.

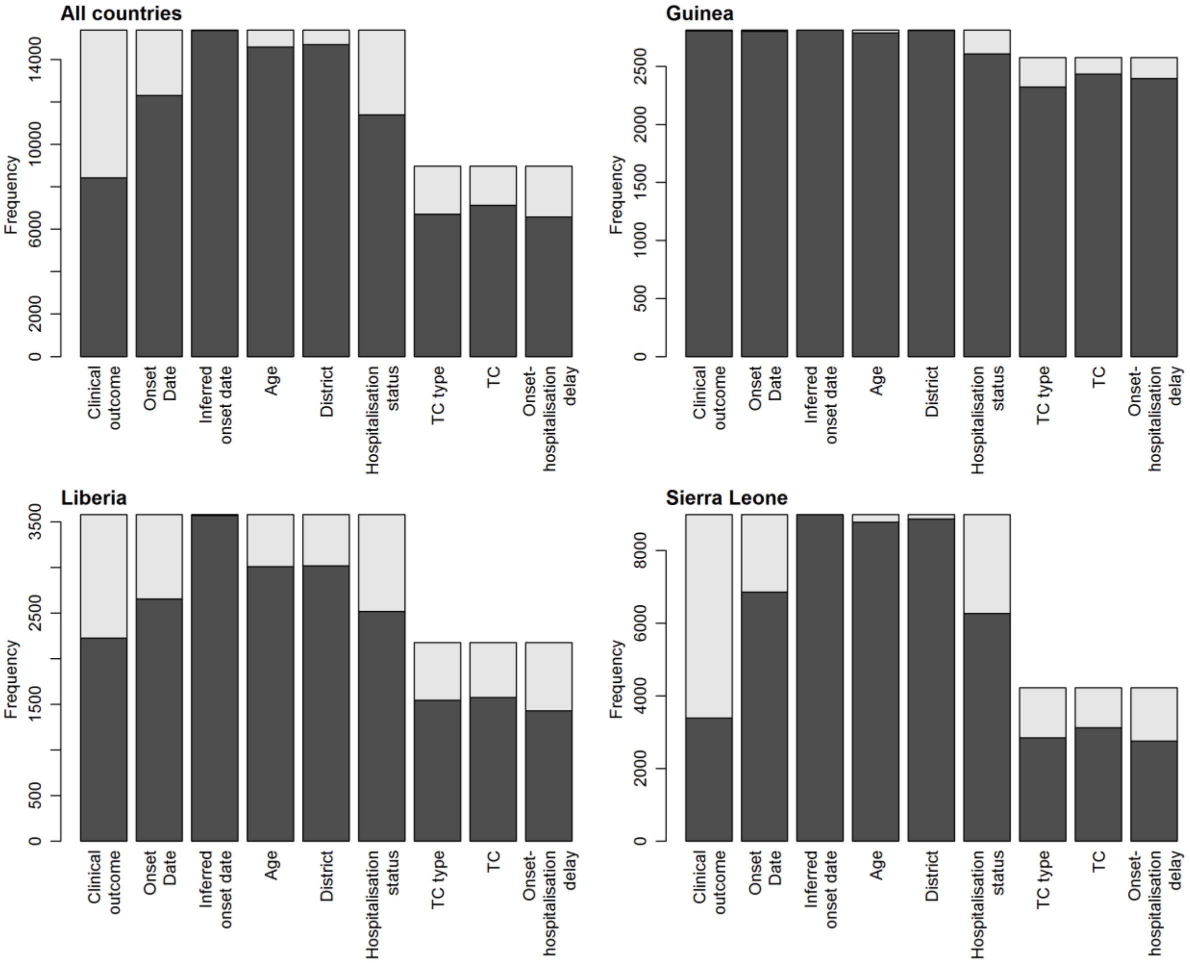

Supplementary Figure 2: Frequency of missing data for key variables among confirmed cases for all countries combined and by country. Dark = recorded, light = missing. The baseline for the three bars on the right is the subset of hospitalised cases. Cases reported retrospectively were excluded.

© The Authors under the terms of the Creative Commons Attribution License http://creativecommons.org/licenses/by/3.0/, which permits unrestricted use, provided the original author and source are credited.

Effect of epidemiological case classification on outcome reporting and CFR estimates

Reporting of clinical outcome varied by country and case definition (Supplementary Figure 3A), with generally the highest reporting rates in confirmed and lowest reporting rates in probable cases. Guinea was an exception, where only a vanishing number of suspected cases were reported, while reporting rates in both confirmed and probable cases were near complete. In Sierra Leone, the outcome was reported slightly more frequently in suspected than confirmed cases. The CFRs based on cases with reported clinical outcome also varied between case definitions, with lowest and most consistent CFRs between countries observed in confirmed cases. In Guinea, death was part of the case definition for probable cases, and therefore the CFR was 100%, while for Liberia and Sierra Leone CFRs were higher in probable and highest in suspected cases as compared to confirmed cases (Supplementary Figure 3B).

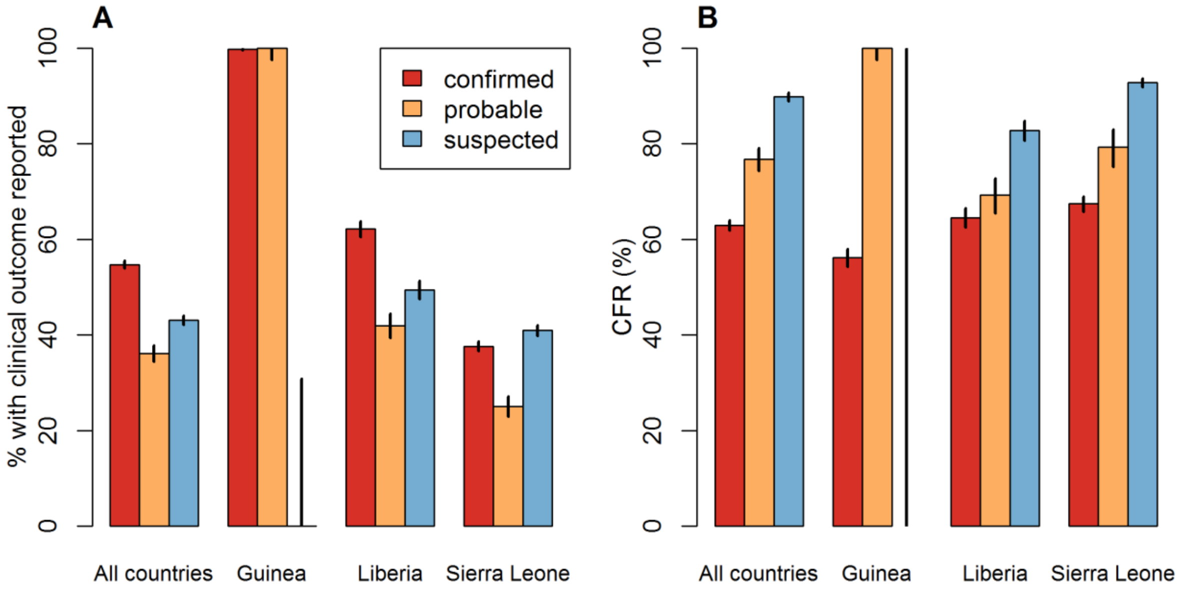

Supplementary Figure 3: A) Percentage of cases with reported definitive clinical outcome and B) CFR, with 95% CI, by country and epidemiological case definition. Cases reported retrospectively were excluded.

Both reporting rates of the clinical outcome and CFR estimates varied not only between countries and case definitions, but also over time as the demands on the health care system changed with incidence (Supplementary Figure 4 and Supplementary Figure 5). While outcome reporting rates were extremely high in Guinea throughout, they decreased in both Liberia and Sierra Leone as case numbers increased, until after the peak. In Liberia very few cases were reported after 2015. In Sierra Leone reporting rates increased as case numbers decreased through 2015. While estimates of the CFR particularly for confirmed cases decreased over time in Guinea and Liberia, in Sierra Leone they increased during the peak until the end of 2014, and then decreased in confirmed cases while they continued to increase to very high values in suspected cases, highlighting a shift in reporting or confirmation practices, or both. It is likely that a higher proportion of suspected cases were truly due to Ebola during the peak than later on when more testing facilities were available while the case load had decreased.

© The Authors under the terms of the Creative Commons Attribution License http://creativecommons.org/licenses/by/3.0/, which permits unrestricted use, provided the original author and source are credited.

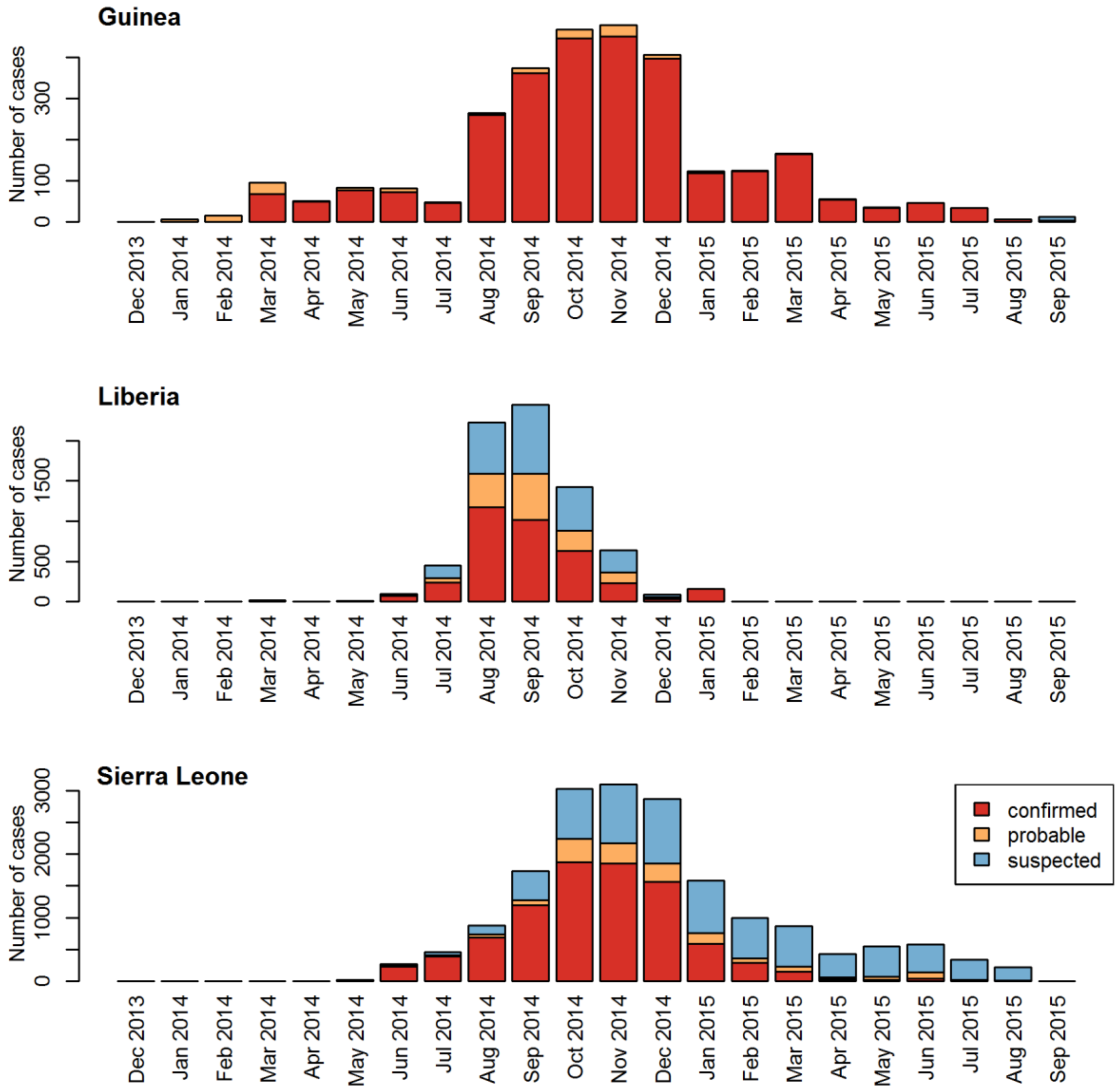

Supplementary Figure 4: Monthly incidence of cases by country and epidemiological case definition, excluding cases reported retrospectively.

© The Authors under the terms of the Creative Commons Attribution License http://creativecommons.org/licenses/by/3.0/, which permits unrestricted use, provided the original author and source are credited.

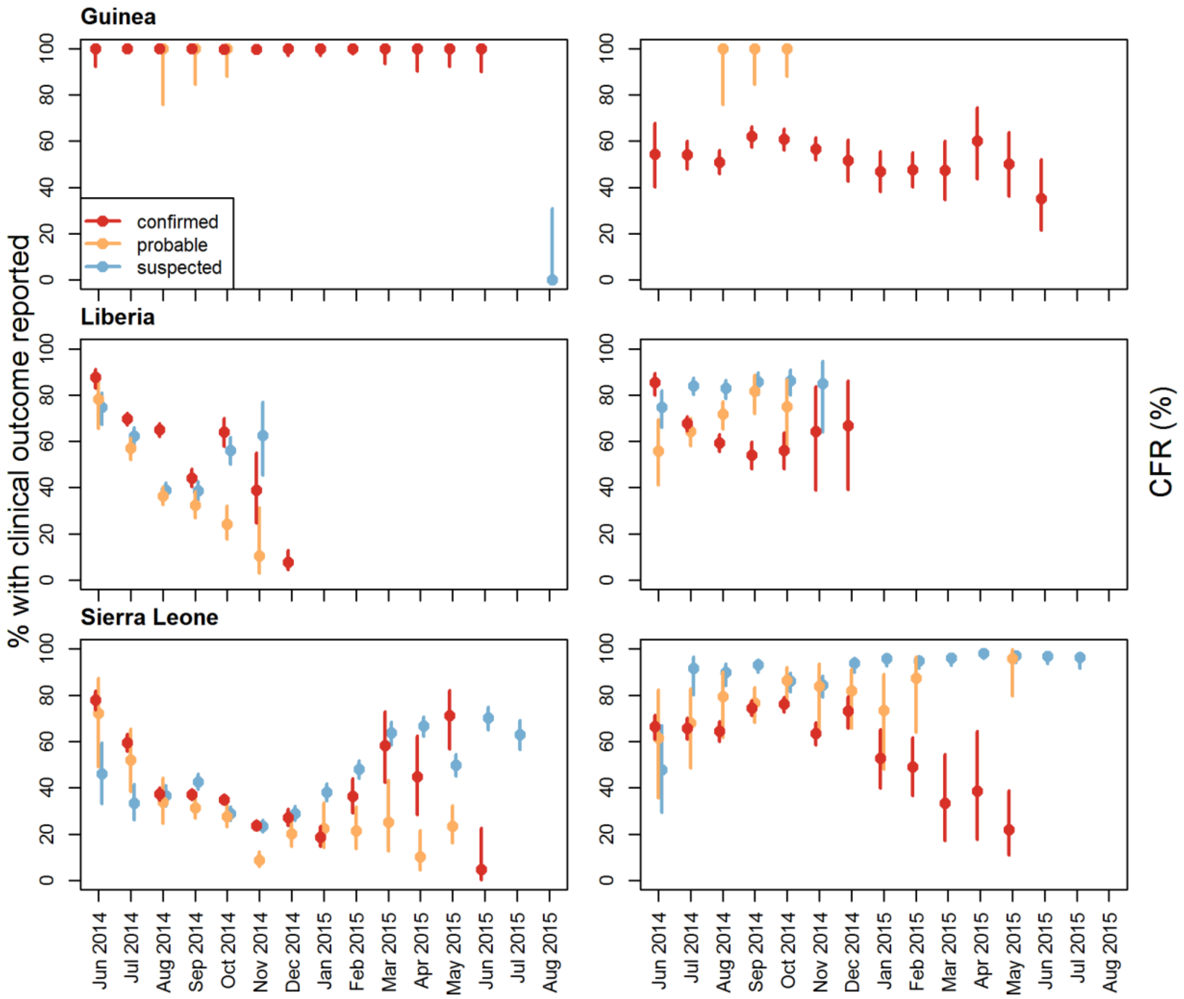

Supplementary Figure 5: Percentage of cases with reported clinical outcome (left) and estimated CFR (right) with 95% CIs by country, month of onset and epidemiological case definition. Only data for months with 10 or more cases are shown. Cases reported retrospectively were excluded.

© The Authors under the terms of the Creative Commons Attribution License http://creativecommons.org/licenses/by/3.0/, which permits unrestricted use, provided the original author and source are credited.

Further results for the multivariable regression analysis

The age-dependence of the CFR shows a striking pattern with lowest CFRs in teenagers and higher mortality at both ends of the age spectrum, which is fitted very well by a 2-parameter model fitting age as a trend with a different slope in individuals under 15 years of age (children) and individuals aged 15 years and above (adults, Supplementary Figure 6). This pattern is evident in all three countries separately, despite a slightly different baseline estimate of the CFR, and while adding country into the model improved the fit significantly, adding an interaction between country and age did not.

In addition to the trends in CFR when stratifying by the covariates used in the multivariable regression (Figure 2 in the main text), Supplementary Figure 7 shows the frequency of cases with reported outcome stratified by each of the covariates, highlighting that most cases were reported in Sierra Leone, in adults aged between 25 and 40 years, during the second half of 2014, and hospitalised in ETUs. While for hospitalised patients the differences in CFR between TC types were relatively small, significance was driven by the sample sizes, and in univariate analyses therefore only TCs of unknown type showed a significantly lower CFR than either ETUs or Health Centres.

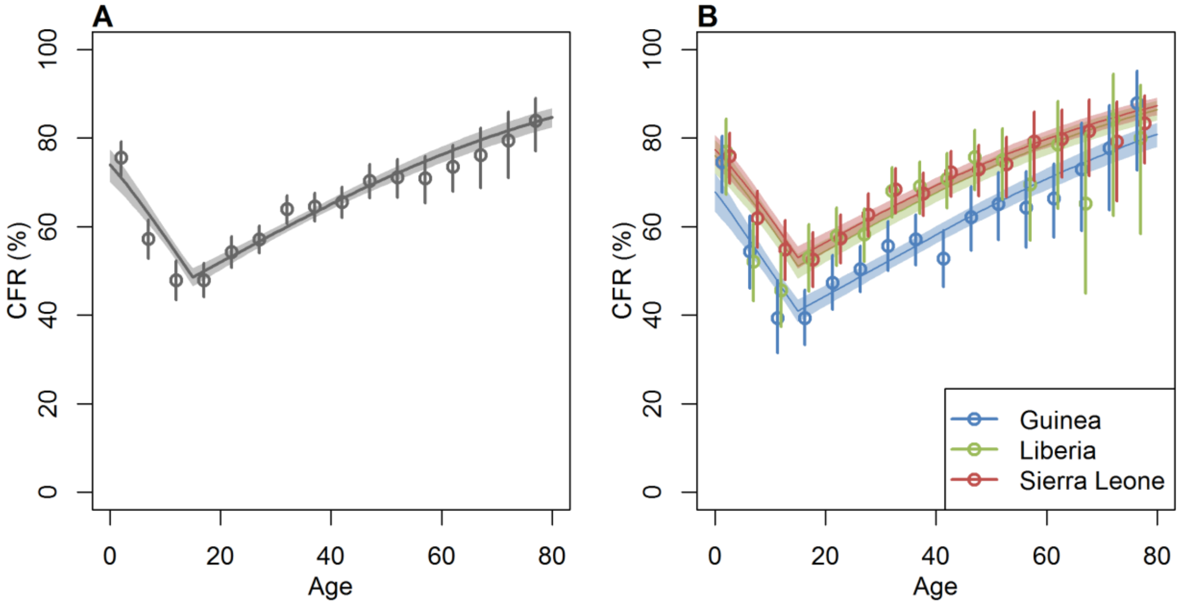

Supplementary Figure 6: CFR among confirmed cases stratified by age for A) all countries and B) by country. Circles and vertical lines show observed CFR (95% CI), solid lines surrounded by shaded areas show predictions (95% CI) from a logistic regression model fitting age as a trend separately in children and adults. Cases reported retrospectively were excluded.

© The Authors under the terms of the Creative Commons Attribution License http://creativecommons.org/licenses/by/3.0/, which permits unrestricted use, provided the original author and source are credited.

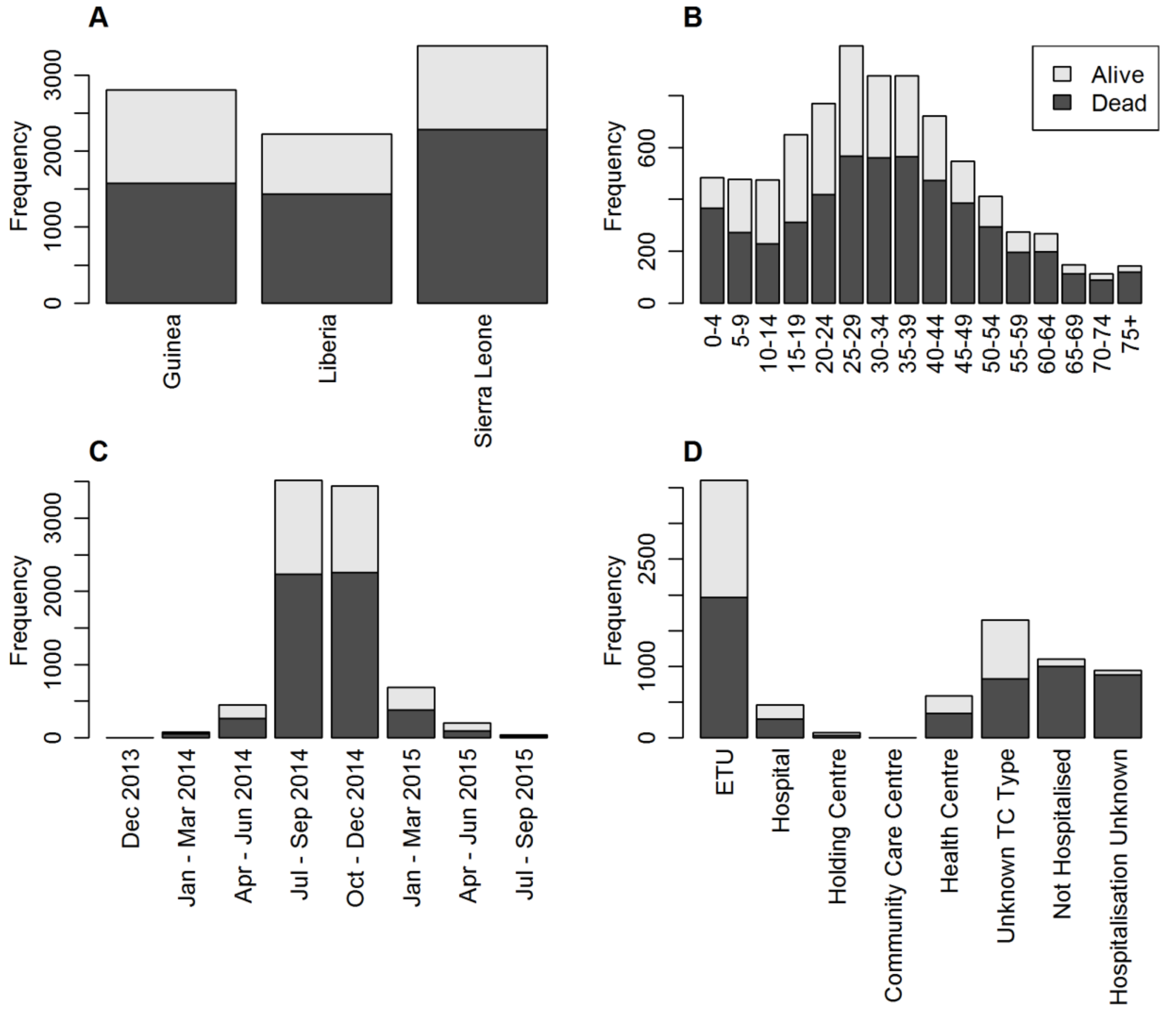

Supplementary Figure 7: Frequency of the covariates considered in the multivariable logistic regression, for confirmed cases, by A) country, B) age, C) inferred onset date and D) TC type. Cases reported retrospectively were excluded.

In multivariable regression modelling the covariates shown in Supplementary Figure 7 are the ones selected in the best-fitting models based on the AIC, for both district- and TC-based analysis, which only included cases for which the district of residence or the TC were recorded, respectively (Supplementary Table 4 and Supplementary Table 5). As the number of cases included in the district-based analysis was considerably larger, including the date of onset as a categorical variable gave a better model fit as indicated by a lower AIC compared to inclusion as a trend, while for the smaller subset of hospitalised cases, the AIC favoured a simpler model with date of onset included as a trend.

© The Authors under the terms of the Creative Commons Attribution License http://creativecommons.org/licenses/by/3.0/, which permits unrestricted use, provided the original author and source are credited.

Supplementary Table 4: Logistic regression coefficients (95% CI) and p-values for the best-fitting model for the CFR by district, fitted to confirmed cases. Coefficients significant at the 95% level are printed in black, those not significant are printed in grey.

	Coefficient	p-value
Intercept	-0.50 (-0.66; -0.34)	<0.001
Age (child in years)	-0.07 (-0.08; -0.05)	<0.001
Age (adult in years)	0.025 (0.021; 0.029)	<0.001
Guinea	0.09 (-0.05; 0.23)	0.2
Liberia	0.24 (0.10; 0.38)	<0.001
Sierra Leone	Reference	--
Onset: Jan - Mar 2014	0.6 (0.1; 1.1)	0.024
Onset: Apr - Jun 2014	-0.1 (-0.3; 0.1)	0.37
Onset: Jul - Sep 2014	0.16 (0.04; 0.28)	0.0079
Onset: Oct - Dec 2014	Reference	--
Onset: Jan - Mar 2015	-0.3 (-0.5; -0.1)	0.0015
Onset: Apr - Jun 2015	-0.7 (-1.0; -0.3)	<0.001
Onset: Jul - Sep 2015	-0.5 (-1.1; 0.2)	0.18
TC type: ETU	Reference	--
TC type: Hospital	0.2 (-0.0; 0.4)	0.073
TC type: Holding Centre	-0.4 (-0.9; 0.1)	0.099
TC type: Community Care Centre	-61 (-295; 273)	0.93
TC type: Health Centre	0.4 (0.2; 0.6)	<0.001
TC type: Unknown TC Type	-0.09 (-0.23; 0.04)	0.18
TC type: Not Hospitalised	2.1 (1.9; 2.4)	<0.001
TC type: Hospitalisation Unknown	2.5 (2.2; 2.7)	<0.001

Supplementary Table 5: Logistic regression coefficients (95% CI) and p-values for the best-fitting model for the CFR by TC, fitted to confirmed cases. Coefficients significant at the 95% level are printed in black, those not significant are printed in grey.

	Coefficient	p-value
Intercept	0.2 (-0.1; 0.4)	0.22
Age (child in years)	-0.08 (-0.09; -0.06)	<0.001
Age (adult in years)	0.026 (0.022; 0.031)	<0.001
Guinea	-0.2 (-0.4; -0.1)	0.0037
Liberia	0.0 (-0.2; 0.2)	0.93
Sierra Leone	Reference	--
Quarter of onset	-0.12 (-0.19; -0.05)	0.0012
TC type: ETU	Reference	--
TC type: Hospital	0.0 (-0.2; 0.3)	0.72
TC type: Holding Centre	-0.7 (-1.2; -0.1)	0.012
TC type: Community Care Centre	-62 (-448; 423)	0.95
TC type: Health Centre	0.2 (0.0; 0.4)	0.049
TC type: Unknown TC Type	0.3 (-0.0; 0.6)	0.09

© The Authors under the terms of the Creative Commons Attribution License http://creativecommons.org/licenses/by/3.0/, which permits unrestricted use, provided the original author and source are credited.

We investigated whether the variation in district- or TC-specific CFR was larger than expected by chance by plotting the CFR observed for each district or TC against the sample size in funnel plots (Supplementary Figure 8). The funnels mark the 95th and 99th centile ranges of the binomial distribution which shrink towards the grand mean with increasing sample size. To illustrate that the observed patterns (panels A and C) show more variation than would be expected purely by chance we re-sampled the outcomes for each district and TC 10 times, and plotted the resulting CFRs (panels B and D). Particularly for districts it is apparent that the observed CFRs show a considerably larger variation above what would be expected by chance in the absence of adjusting for co-variates. However, when adjusting for the covariates included in the multivariable logistic regression models identified above, not all districts identified as outliers lay outside the funnel (Figure 3 in the main text).

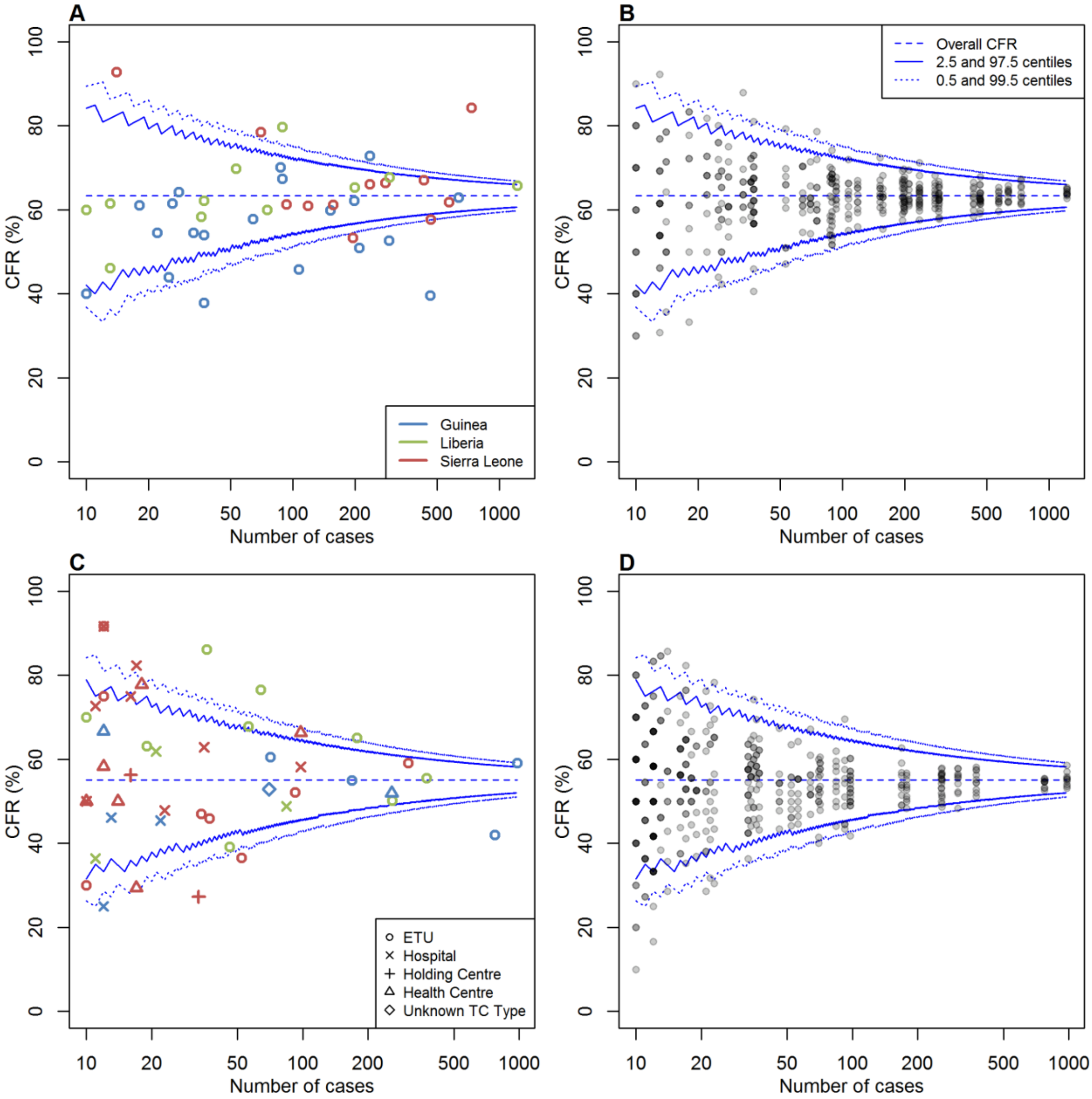

Supplementary Figure 8: A) CFR by district, B) re-sampled CFR by district, C) CFR by TC, D) re-sampled CFR by TC. For each district or TC, outcomes were re-sampled 10 times and the CFRs plotted in semi-transparent circles. Darker circles correspond to multiple realisations for a particular district or TC yielding the same CFR.

© The Authors under the terms of the Creative Commons Attribution License http://creativecommons.org/licenses/by/3.0/, which permits unrestricted use, provided the original author and source are credited.

Sensitivity Analysis: Main results for confirmed and probable cases

While we restricted the main analysis to confirmed cases only as the reporting was most consistent among these and therefore the biases in CFR estimates would be smaller, here we repeated these analyses for confirmed and probable cases to investigate sensitivity to this restriction. The overall CFR was significantly higher for probable than confirmed cases (76.8% (95% CI 74.3% – 79.1%) for probable cases only compared to 62.9% (95% CI: 61.9% – 64.0%) for confirmed cases, respectively). However, the fairly small number of probable cases means that the overall CFR estimate for confirmed and probable cases was similar to that of the confirmed cases only, at 64.6% (95% CI 63.6% – 65.6%).

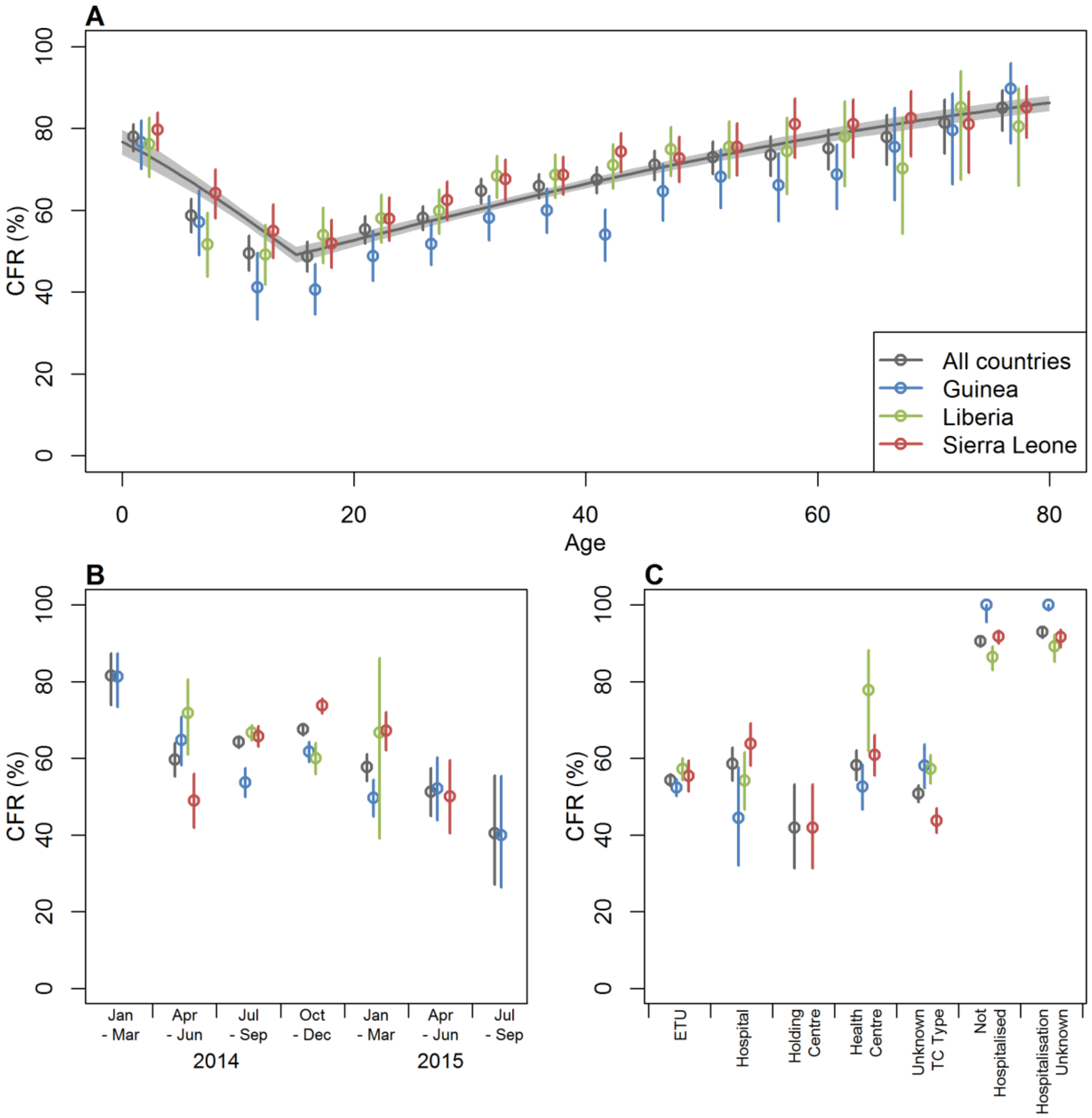

Supplementary Figure 9: CFR by A) age in 5 year age bands, B) quarter, C) Treatment Centre type, for all countries combined and by country for confirmed and probable cases, with 95% CI. Only strata with 10 or more confirmed and probable cases are shown. Note that there were no Community Care Centres with 10 or more cases with reported clinical outcome. A) The solid black line shows the prediction from a logistic regression model with age fitted as a linear trend in children and adults separately with 95% CI (grey area), shown for all countries combined.

© The Authors under the terms of the Creative Commons Attribution License http://creativecommons.org/licenses/by/3.0/, which permits unrestricted use, provided the original author and source are credited.

The relationships with the covariates considered were very similar for confirmed and probable as compared to confirmed cases (Supplementary Figure 9 and Figure 2 in the main text, respectively).

For confirmed and probable cases in the district-based analysis, the AIC favoured a slightly simpler multivariable logistic regression model than for confirmed cases only, not including country as a covariate (Supplementary Table 6 vs Supplementary Table 4). Symptom onset was fitted as a categorical variable as for confirmed cases only, and the same strata were significant. Among TC types only Community Care Centres were non-significant, while the differences between countries may have been captured by a different composition in TC types between countries in this analysis, leading to more significant strata here compared to the analysis for confirmed cases only. In this analysis we also explored the case classification (confirmed vs probable) as a potential covariate. The model including case classification in addition to the same variables included in the model fitted to confirmed cases only had an AIC that was higher by only 1.78 compared to the selected model, indicating only a moderate preference for the simpler model.

Supplementary Table 6: Logistic regression coefficients (95% CI) and p-values for the best-fitting model for the CFR by district, fitted to confirmed and probable cases. Coefficients significant at the 95% level are printed in black, those not significant are printed in grey.

	Coefficient	p-value
Intercept	-0.38 (-0.50; -0.27)	<0.001
Age (child in years)	-0.071 (-0.086; -0.056)	<0.001
Age (adult in years)	0.025 (0.021; 0.029)	<0.001
Onset: Jan - Mar 2014	1.0 (0.5; 1.5)	<0.001
Onset: Apr - Jun 2014	-0.1 (-0.3; 0.1)	0.28
Onset: Jul - Sep 2014	0.12 (0.01; 0.22)	0.032
Onset: Oct - Dec 2014	Reference	--
Onset: Jan - Mar 2015	-0.3 (-0.5; -0.1)	0.0019
Onset: Apr - Jun 2015	-0.6 (-0.9; -0.3)	<0.001
Onset: Jul - Sep 2015	-0.5 (-1.2; 0.1)	0.13
TC type: ETU	Reference	--
TC type: Hospital	0.2 (0.0; 0.4)	0.049
TC type: Holding Centre	-0.6 (-1.0; -0.1)	0.021
TC type: Community Care Centre	-61 (-295; 272)	0.93
TC type: Health Centre	0.3 (0.1; 0.5)	0.0015
TC type: Not Hospitalised	2.0 (1.9; 2.2)	<0.001
TC type: Hospitalisation Unknown	2.4 (2.1; 2.7)	<0.001

For TC-based analysis the model selected for confirmed and probable cases was the same as for confirmed cases only, not including the case classification although this was explored as well. The patterns of which particular strata were significant were also the same as for confirmed cases only (Supplementary Table 7 vs Supplementary Table 5).

© The Authors under the terms of the Creative Commons Attribution License http://creativecommons.org/licenses/by/3.0/, which permits unrestricted use, provided the original author and source are credited.

Supplementary Table 7: Logistic regression coefficients (95% CI) and p-values for the best-fitting model for the CFR by TC, fitted to confirmed and probable cases. Coefficients significant at the 95% level are printed in black, those not significant are printed in grey.

	Coefficient	p-value
Intercept	0.1 (-0.1; 0.4)	0.27
Age (child in years)	-0.08 (-0.10; -0.06)	<0.001
Age (adult in years)	0.026 (0.022; 0.031)	<0.001
Guinea	-0.24 (-0.40; -0.08)	0.0026
Liberia	-0.0 (-0.2; 0.1)	0.59
Sierra Leone	Reference	--
Quarter of onset	-0.11 (-0.18; -0.04)	0.0016
TC type: ETU	Reference	--
TC type: Hospital	0.1 (-0.1; 0.3)	0.45
TC type: Holding Centre	-0.7 (-1.2; -0.2)	0.0073
TC type: Community Care Centre	-62 (-448; 423)	0.95
TC type: Health Centre	0.2 (0.0; 0.4)	0.048
TC type: Unknown TC Type	0.3 (-0.0; 0.6)	0.058

In alignment with the above results, the patterns of outliers identified in the funnel plots and the statistical analysis adjusting for covariates were somewhat different for districts compared to the analysis for confirmed cases only (Supplementary Figure 10A and Supplementary Table 8), with nine outlying districts identified here, six of which were also among the eight identified when considering confirmed cases only. For TC-based analysis, the outliers identified were the same as for confirmed cases only (Supplementary Figure 10B and Supplementary Table 9).

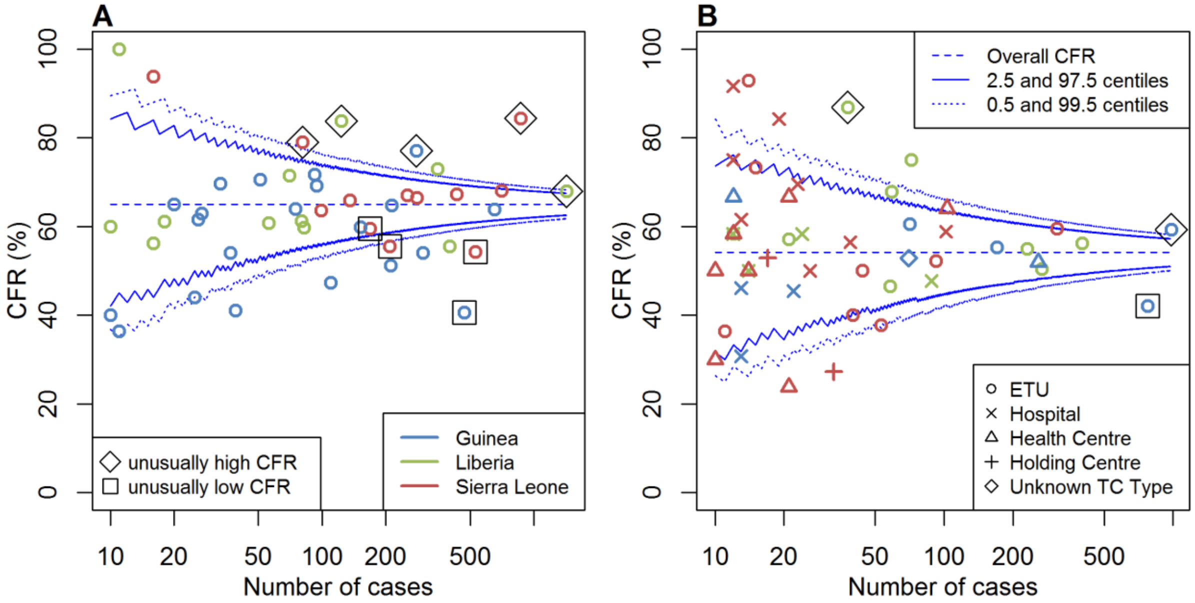

Supplementary Figure 10: Funnel plot of the CFR by A) district of residence and B) TC, for confirmed and probable cases. Only districts and TCs with 10 or more confirmed and probable cases are included. Districts and TCs with significantly high or low CFR after adjusting for covariates are marked with diamonds and squares, respectively. Blue solid and dotted lines show the 95% and 99% binomial confidence intervals, respectively, for each sample size assuming the mean CFR of the cases included in these analyses (dashed blue line).

© The Authors under the terms of the Creative Commons Attribution License http://creativecommons.org/licenses/by/3.0/, which permits unrestricted use, provided the original author and source are credited.

Supplementary Table 8: Districts with significantly lower or higher CFR among confirmed and probable cases than expected based on case mix, adjusting for age, quarter of onset and TC type.

Country	District	N	Observed CFR (95% CI)	Expected CFR* (95% CI)	Direction	p-value
Guinea	CONAKRY	470	40.6 (36.3 - 45.1)	54.9 (50.3 - 59.3)	low	<0.001
Sierra Leone	KAMBIA	168	59.5 (52 - 66.7)	79.1 (72.3 - 84.5)	low	<0.001
Sierra Leone	MOYAMBA	209	55.5 (48.7 - 62.1)	67.9 (61.3 - 73.8)	low	0.0033
Sierra Leone	KAILAHUN	529	54.3 (50 - 58.5)	61.7 (57.5 - 65.8)	low	0.018
Liberia	MONTSERRADO	1429	67.9 (65.5 - 70.3)	64.1 (61.6 - 66.5)	high	<0.001
Guinea	GUECKEDOU	279	77.1 (71.8 - 81.6)	61.8 (56 - 67.3)	high	<0.001
Sierra Leone	WESTERN	865	84.4 (81.8 - 86.7)	76.3 (73.4 - 79)	high	<0.001
Liberia	BOMI	123	83.7 (76.2 - 89.2)	67.5 (58.8 - 75.2)	high	<0.001
Sierra Leone	TONKOLILI	81	79 (68.9 - 86.5)	62.7 (51.8 - 72.4)	high	0.017

*expected based on case mix adjusting for age, quarter of onset (fitted as a categorical variable) and TC type.

Supplementary Table 9: Treatment centres with significantly lower or higher CFR among confirmed and probable cases than expected based on case mix, adjusting for age, quarter of onset and TC type.

Country	Treatment centre†	N	Observed CFR (95% CI)	Expected CFR* (95% CI)	Direction	p-value
Guinea	Conakry 2	775	42.1 (38.6 - 45.6)	55.1 (51.5 - 58.5)	low	<0.001
Guinea	Gueckedou 1	985	59.3 (56.2 - 62.3)	46.7 (43.6 - 49.9)	high	<0.001
Liberia	Montserrado 65	38	86.8 (72.7 - 94.2)	58.4 (42.7 - 72.6)	high	0.002

†Treatment centre names were anonymised within district.

*expected based on case mix adjusting for country, age, quarter of onset (fitted as trend) and TC type.

Broadly, the analysis of confirmed and probable cases gave very similar results to that of confirmed cases only even though clearly probable cases differed from confirmed cases in important ways, including outcome reporting and mortality. While for the district based analysis this had an effect on the best fitting model selected and the outliers identified, for TC-based analysis both the model and identified outlying TCs were identical between both analyses. This is in line with the fact that probable cases make up 17% of confirmed and probable cases overall, but only around 12% of the hospitalised confirmed and probable cases.

© The Authors under the terms of the Creative Commons Attribution License http://creativecommons.org/licenses/by/3.0/, which permits unrestricted use, provided the original author and source are credited.
